# Targeting CD47 in Anaplastic Thyroid Carcinoma Enhances Tumor Phagocytosis by Macrophages and Is a Promising Therapeutic Strategy

**DOI:** 10.1089/thy.2018.0555

**Published:** 2019-07-17

**Authors:** Christian M. Schürch, Matthias A. Roelli, Stefan Forster, Marie-Hélène Wasmer, Frido Brühl, Renaud S. Maire, Sergio Di Pancrazio, Marc-David Ruepp, Roland Giger, Aurel Perren, Anja M. Schmitt, Philippe Krebs, Roch-Philippe Charles, Matthias S. Dettmer

**Affiliations:** ^1^ Institute of Pathology, University of Bern, Bern, Switzerland.; ^2^ Baxter Laboratory for Stem Cell Biology, Department of Microbiology and Immunology, Stanford University School of Medicine, Stanford, California.; ^3^ Institute of Biochemistry and Molecular Medicine, University of Bern, Bern, Switzerland.; ^4^ Graduate School for Cellular and Biomedical Sciences, University of Bern, Bern, Switzerland.; ^5^ Department of BioMedical Research, University of Bern, Bern, Switzerland.; ^6^ Department of Chemistry and Biochemistry, University of Bern, Bern, Switzerland.; ^7^ United Kingdom Dementia Research Institute Centre, Department of Basic and Clinical Neuroscience, Institute of Psychiatry, Psychology and Neuroscience, Maurice Wohl Clinical Neuroscience Institute, King's College London, London, United Kingdom.; ^8^ Department of Oto-Rhino-Laryngology, Head and Neck Surgery, Inselspital, Bern University Hospital and University of Bern, Bern, Switzerland.

**Keywords:** anaplastic thyroid carcinoma, CD47, immune checkpoints, phagocytosis, programmed cell death 1, tumor-associated macrophages

## Abstract

***Background:*** Anaplastic thyroid carcinoma (ATC) is one of the most aggressive human cancers, with a median survival of only three to six months. Standard treatment options and even targeted therapies have so far failed to improve long-term overall survival. Thus, novel treatment modalities for ATC, such as immunotherapy, are urgently needed. CD47 is a “don't eat me” signal, which prevents cancer cells from phagocytosis by binding to signal regulatory protein alpha on macrophages. So far, the role of macrophages and the CD47–signal regulatory protein alpha signaling axis in ATC is not well understood.

***Methods:*** This study analyzed 19 primary human ATCs for macrophage markers, CD47 expression, and immune checkpoints by immunohistochemistry. ATC cell lines and a fresh ATC sample were assessed by flow cytometry for CD47 expression and macrophage infiltration, respectively. CD47 was blocked in phagocytosis assays of co-cultured macrophages and ATC cell lines. Anti-CD47 antibody treatment was administered to ATC cell line xenotransplanted immunocompromised mice, as well as to tamoxifen-induced ATC double-transgenic mice.

***Results:*** Human ATC samples were heavily infiltrated by CD68- and CD163-expressing tumor-associated macrophages (TAMs), and expressed CD47 and calreticulin, the dominant pro-phagocytic molecule. In addition, ATC tissues expressed the immune checkpoint molecules programmed cell death 1 and programmed death ligand 1. Blocking CD47 promoted the phagocytosis of ATC cell lines by macrophages *in vitro*. Anti-CD47 antibody treatment of ATC xenotransplanted mice increased the frequency of TAMs, enhanced the expression of macrophage activation markers, augmented tumor cell phagocytosis, and suppressed tumor growth. In double-transgenic ATC mice, CD47 was expressed on tumor cells, and blocking CD47 increased TAM frequencies.

***Conclusions:*** Targeting CD47 or CD47 in combination with programmed cell death 1 may potentially improve the outcomes of ATC patients and may represent a valuable addition to the current standard of care.

## Introduction

Anaplastic thyroid carcinoma (ATC) is one of the most aggressive solid tumors in humans, with a case fatality rate approaching 100% and a median survival of three to six months. With an incidence of one to two per million people per year, ATC represents an orphan disease that accounts for <10% of all thyroid cancers worldwide (1,2). However, ATCs are most often locally advanced or metastatic upon presentation and are therefore responsible for a substantial fraction of thyroid cancer-related deaths (3). Due to the rarity of this tumor, the management of patients with ATC is based on clinical experience and published case series. Depending on the disease stage and local resectability, surgery combined with radiotherapy and chemotherapy represents the current treatment of choice. For inoperable and metastatic tumors, aggressive therapy, consisting of tumor debulking surgery, before or after local radiation and systemic therapy, or palliative care are the only options (4). Radioactive iodine therapy is not effective because ATCs do not concentrate iodine (5). Survival rates have remained unchanged in the last 30 years. Generally, regardless of treatment strategy, the outcomes of ATC are very poor. Therefore, all ATC patients should be considered candidates for clinical trials investigating novel experimental approaches including targeted therapies and immunotherapies (6,7).

Recently, therapies aiming at modulating the immune system have led to impressive results in various advanced human tumors (8). In particular, immune checkpoint inhibitors—therapeutic antibodies that enhance antitumoral T-cell responses by blocking inhibitory molecules such as cytotoxic T lymphocyte antigen 4 (CTLA-4) and programmed cell death 1 (PD-1)—have proven very effective and have even resulted in long-term remission in patients with metastatic cancers (9,10). In addition, accumulating evidence points toward a very important role for innate immune cells, including natural killer (NK) cells and tumor-associated macrophages (TAMs), in the biology of malignant neoplasms (11–13). TAMs are considered a double-edged sword in cancer, showing either pro-tumorigenic or tumor-suppressive functions depending on their activation state (14). Because TAMs account for a major fraction of the immune infiltrate in many tumors, reprogramming these cells toward an antitumoral state by enhancing their capacity to phagocytose tumor cell (“programmed cell removal”) is an attractive strategy in cancer immunotherapy (15).

CD47 is an immunoglobulin family member protein overexpressed on the surface of many cancer types, including leukemias, lymphomas, and solid tumors (16–23). CD47 acts as a “don't eat me” signal by binding to signal regulatory protein alpha (SIRPα) on macrophages and thereby inhibiting phagocytosis of target cells (24–27). Consequently, CD47 blockade enhances tumor cell phagocytosis by TAMs, improves survival in various murine cancer models, and is currently being tested in multiple clinical trials (16,17,19–22,28).

The present study investigated the role of TAMs and CD47 in ATC. It demonstrates that primary human ATCs are heavily infiltrated by TAMs and express significant levels of CD47 and calreticulin, the dominant pro-phagocytic molecule (29), as well as the immune checkpoint molecules PD-1 and PD-1 ligand 1 (PD-L1). Blocking CD47 promoted the phagocytosis of ATC cell lines by macrophages *in vitro* and *in vivo* and increased the frequency of TAMs in ATC xenografts and in a double-transgenic ATC mouse model. Taken together, these data reveal that targeting of CD47 may provide a novel therapeutic strategy for ATC patients for whom effective therapeutic options are otherwise currently very limited.

## Methods

### Patient samples

Formalin-fixed, paraffin-embedded (FFPE) tissues from 19 patients (14 females; *M*_age_ ± SEM = 72.5 ± 2 years) with a diagnosis of ATC between 2005 and 2018 were identified in the archives of the Institute of Pathology, University of Bern (Bern, Switzerland). Cases were reviewed in detail by three board-certified surgical pathologists with a special interest in thyroid pathology (A.P., A.M.S., and M.S.D.), and were reclassified/restaged according to the 2017 World Health Organization Classification of Tumors of Endocrine Organs and the 2017 American Joint Committee on Cancer/Union for International Cancer Control tumor-node-metastasis guidelines (30–32). Patient characteristics are detailed in Table 1 and Supplementary Table S1. The study was approved by the local Ethics Committee of the Canton of Bern (KEK 200/14, KEK 2018-01502). A fresh sample for fluorescence-activated cell sorting (FACS) analysis was obtained after surgery and diagnosis of ATC in frozen sections, with written informed consent from the patient.

**Table 1.** Patient Characteristics

*Clinical characteristics at diagnosis*	
Age (mean ± SEM), years	72.5 ± 2
Median, years	72
Range, years	58–86
Female/male ratio (*n*)	2.8 (14/5)
*TNM classification and tumor staging,* n	
Primary tumor:	
pT3a	3
pT4a	16
Regional lymph nodes:	
pN0	2
pN1	9
pNX	8
Distant metastases:	
M0	3
M1	11
MX	5
Resection status:	
R0	1
R1/R2	13/5
Site of distant metastases:	
Lung	6
Other	7
Unknown	4
AJCC stage:	
IVB	7
IVC	12
*Therapy,* n	
Thyroidectomy and/or tumor debulking	19
Neck dissection	9
Radiotherapy	8
Chemotherapy	5
Radioiodine therapy	1
Comfort/palliative therapy	7
*Survival*	
Median (months)	3.5
Range (months)	0.9–61
<6 months, *n*	12
12–24 months, *n*	3
Alive with stable disease, *n* (months after diagnosis)	1 (61)
Lost to follow-up, *n* (months after diagnosis)	3 (1.9, 1.9, 18.1)
*Cause of death,* n	
Tumor related	13
Non-tumor related	1
Unknown	1

Further details are listed in Supplementary Table S1.

### Immunohistochemistry

All sections were cut to 2 μm thickness. Hematoxylin and eosin–stained sections were obtained from each FFPE block. Immunohistochemistry (IHC) staining of full slides from FFPE blocks was performed on a Leica BOND RX automated immunostainer using Bond primary antibody diluent and Bond Polymer Refine DAB detection kit according to the manufacturer's instructions (Leica Biosystems). Details on antibodies, clones, manufacturers, and staining conditions for IHC are listed in Supplementary Table S2. Analysis and interpretation of the staining results were performed by two board-certified surgical pathologists (C.M.S and M.S.D.) and one pathologist in training (S.F.) in accordance with the “REporting recommendations for tumor MARKer prognostic studies” guidelines (33). Tumor cells were morphologically identified by cell size, shape, and nuclear configuration. CD47 staining in tumor cells was classified microscopically as 0 (absence of any membranous or cytoplasmic staining), 1+ (weak or incomplete membranous and/or cytoplasmic staining), 2+ (complete membranous staining of intermediate intensity), and 3+ (complete membranous staining of strong intensity). The calreticulin staining pattern was mostly granular and cytoplasmic and was classified microscopically as 0–3+. For CD68, CD163, PD-1, and PD-L1 staining, the positive cell frequencies were estimated by microscopy and were quantified by QuPath analysis, as described below. The concordance of microscopical estimation and QuPath quantification was in the range of ±10% for all cases, except for PD-1 and PD-L1 staining in 7 and 10 cases, respectively, which could not be evaluated adequately by automated QuPath analysis due to the predominantly weak membranous staining pattern. Therefore, for PD-1 and PD-L1 staining, only the values from microscopical estimation were used. All results are detailed in Supplementary Table S1.

### Slide digitization, cell annotation, and QuPath analysis

Slides were scanned using an Aperio Scanscope CS digital slide scanner (Leica Biosystems) and analyzed using QuPath software v0.1.2. (34). For each sample, a selected and defined tumor area (at least 1 mm^2^) was analyzed. For detection of macrophages (CD68, CD163), T cells (CD3, CD4, CD8), granulocytes (CD15), NK cells (CD56), plasmacytoid dendritic cells (CD123), vasculature (CD31), as well as PD-1^+^ and PD-L1^+^ cells, the QuPath positive cell detection algorithm was used with the following setup parameters: detection image, hematoxylin OD for CD68, CD163, PD-1, and PD-L1; optical density sum for CD3, CD4, CD8, CD15, CD56, CD123, and Ki-67; requested pixel size, 0.5 μm; nucleus parameters—background radius 8 μm, median filter radius 0 μm, sigma 2.0 μm, minimum area 10 μm^2^, and maximum area 400 μm^2^; intensity parameters—threshold 0.02, maximum background intensity 2.0, split by shape yes, exclude DAB (membrane staining) no; cell parameters—cell expansion 3 μm include cell nucleus yes; general parameters—smooth boundaries yes, make measurements yes; and intensity threshold parameters—score compartment: cell, DAB OD mean, threshold 1 + 0.2, single threshold yes. For Ki-67 staining, the scoring compartment in intensity threshold parameters was switched to nucleus: DAB OD mean. For samples showing a stronger background staining (especially CD163 IHC), setup intensity parameters were modified as follows: intensity threshold parameters—score compartment: cell, DAB OD mean, threshold 1 + 0.2, threshold 2 + 0.4, threshold 3 + 0.5, single threshold no. The quality of segmentation and positive and negative cell detection was visually analyzed and confirmed for each case.

### Cell lines

The human ATC cell lines 8505C (35), 8305C (35), HTH-104 (36), BHT-101 (37), CAL-62 (38), C643 (39), and SW-1736 (40) were obtained from Prof. Dr. Martin Walter (Department of Nuclear Medicine, Inselspital, Bern University Hospital, Bern, Switzerland), and have been described before. The human ATC cell line OCUT-2 (41) was obtained from R.-P.C. and has been described before.

### Mice

NOD.Cg-*Prkdc^scid^ Il2rg^tm1WjI^*/SzJ (NSG) mice (42) were purchased from Charles River Laboratories. *Braf^CA/+^; Pik3ca^Lat/+^; ThyroglobulinCre^ERT2^* (Thyro-DT) mice from a mixed FVB/C57BL6/F129 background have been previously described (43). Mice were housed under specific pathogen-free conditions in isolated ventilated cages on a 12-hour/12-hour cycle of light and dark, fed *ad libitum*, and regularly monitored for pathogens. All mouse experiments were licensed by the Canton of Bern and were performed in compliance with Swiss Federal legislation.

### *In vitro* phagocytosis assay

The *in vitro* phagocytosis assay was performed as described before (23). Buffy coats and human serum were obtained from the Swiss Blood Bank (Interregionale Blutspende SRK, Bern, Switzerland) under the signed consent of the donors and in agreement with local legislation. Peripheral blood mononuclear cells (PBMCs) were enriched from buffy coats by density centrifugation using Lympho Spin Medium (pluriSelect). Monocytes were isolated from PBMCs using the EasySep Human CD14 Positive Selection Kit II (Stemcell Technologies) according to the manufacturer's instructions. Monocytes (4–5 × 10^6^ per well) were differentiated into macrophages for seven days at 37°C, 5% CO_2_, on six-well tissue culture plates in Iscove's Modified Dulbecco's Medium supplemented with 10% human serum, 1% L-glutamine, and 1% penicillin/streptomycin. 8505C cells were harvested using non-enzymatic cell dissociation buffer (Sigma–Aldrich), washed in phosphate-buffered saline (PBS) three times, and then labeled with 5(6)-carboxyfluorescein diacetate *N*-succinimidyl ester (CFSE) in PBS at a final concentration of 20 μM. Macrophages were starved in serum-free medium for two hours, and 1 × 10^6^ CFSE-labeled 8505C cells per well were added. Cells were co-cultured for two hours in the presence of 10 μg/mL mouse anti-human CD47 (clone B6H12.2; Thermo Fisher Scientific) or isotype control (mouse IgG1; Thermo Fisher Scientific). Then, wells were imaged at 20 × magnification on a digital inverted fluorescence microscope (EVOS™ FL imaging system; Thermo Fisher Scientific). Phase contrast and green fluorescent protein (GFP) overlay images were exported, and the phagocytosis index and CFSE^+^ cells per macrophage were manually determined by two pathologists in training (S.F. and F.B.) in an independent fashion for each experiment. After microscopic imaging, cells were harvested using trypsin/EDTA solution (Sigma–Aldrich), stained for anti-human CD45 and CD14, and analyzed by FACS.

### Stable GFP transduction of 8505C cells

293T cells were cultured in Dulbecco's modified Eagle's medium (DMEM)/F12 supplemented with 10% tetracycline-free fetal calf serum (FCS), penicillin (100 IU/mL), and streptomycin (100 μg/mL), and grown to 80% confluency in T150 flasks. The pLVX-EF1α-TS-EGFP-IRES-Puro vector was cloned by introducing a TS-EGFP DNA string (GeneArt^®^ Elements™; Thermo Fisher Scientific) into the *EcoR*I and *BamH*I sites of the pLVX-EF1α-IRES-Puro vector (Takara Bio, Inc./Clontech). The construct was verified by sequencing. For transient transfection of 293T cells, 20 μg pLX-EF1α-TS-EGFP-IRES-Puro and 60 μL Lenti-X packaging mix (GE Healthcare) were mixed with 240 μL Dogtor (OZ Biosciences) in a total volume of 500 μL Opti-MEM (Thermo Fisher Scientific) followed by 20 minutes of complex formation at room temperature and incubation with the cells. Twenty-four hours post transfection, the medium was exchanged. Lentiviral supernatants were harvested at 48, 72, and 96 hours post transfection and filtered through a 0.45 μm PES filter, followed by sixfold concentration with Lenti-X Concentrator (Takara Bio, Inc./Clontech) according to the manufacturer's instructions. 8505C cells cultured in T25 flasks in RPMI-1640 supplemented with 10% tetracycline-free FCS, penicillin/streptomycin, and 1% L-glutamine were transduced three times with 1 mL viral supernatant at a 1:5 dilution. Forty-eight hours post transduction, cells were selected with puromycin at a final concentration of 3 μg/mL for nine days. Finally, GFP^hi^-expressing cells were purified by FACS sorting.

### Tumor cell injection, tumor measurement, and treatment of NSG mice

Parental or stably GFP^hi^-expressing 8505C cells (4–5 × 10^6^) were injected subcutaneously (s.c.) into the flanks of six-week-old female NSG mice. Starting three days after tumor cell injection, mice were treated with 500 μg anti-human CD47 monoclonal antibody (mAb; clone B6H12; BioXCell) or mouse IgG1 isotype control mAb (clone MOPC-21; BioXCell) by intraperitoneal (i.p.) injection twice a week. Tumor growth was measured with a caliper twice a week, and tumor volumes were calculated by the formula *V* = (π × width × length × height)/6. After five to six weeks, mice were sacrificed by CO_2_ inhalation followed by cervical dislocation, and tumors were excised, measured, and weighed.

### Tumor induction, tumor measurement, and treatment of Thyro-DT mice

Tumors were induced in Thyro-DT mice by daily i.p. injections of 1 mg tamoxifen diluted in 100 μL peanut oil (Sigma–Aldrich) on five consecutive days. To monitor tumor development, tumors were measured by ultrasound every three weeks. Mice were anesthetized using 5 μL/g of body weight of a mixture of 0.1 mg/mL medetomidine, 0.5 mg/mL midazolam, and 5 μg/mL fentanyl in 0.9% NaCl (Sigma–Aldrich) by i.p. injection. The fur around the neck was epilated with Veet^®^ hair removal cream. Images were acquired with an ESAOTE MyLab Five ultrasound device equipped with an 18 MHz LA455 Probe (Siemens). After imaging, anesthesia was reversed with 10 μL/g body weight of a mixture of 0.25 mg/mL atipamezole, 5 μg/mL flumazenil, and 20 μg/mL naloxone in 0.9% NaCl (Sigma–Aldrich) by s.c. injection. Ultrasound images were analyzed using ImageJ software. After three months of tumor growth, tumor-bearing mice were evenly divided into two groups according to tumor sizes. All mice weighed between 20 and 30 g at the start of the experiment. Mice were treated with either 500 μg (5 mg/mL) of anti-mouse/human/rat CD47 mAb (clone MIAP410; BioXCell; treatment group) or 100 μl *InVivo*Pure, pH 7.0, dilution buffer (BioXCell; control group) by i.p. injection twice a week. During treatment, ultrasound measurements of tumor size were performed every two weeks. Tumor size was approximated by the biggest area in cross-section found for each tumor. Continuous measurements were normalized to the measurement at the start of treatment to create tumor growth curves for each individual mouse.

### Perfusion and organ excision

For sample preparation, Thyro-DT mice were anesthetized with 10 mg/mL ketamine and 1.6 mg/mL xylazine at a dose of 10 μL/g body weight by i.p. injection. When mice were unresponsive to mechanical stimuli, the thoracic and abdominal cavities were opened. The heart was punctured on the right side, and PBS supplemented with 137 mM NaCl, 2.7 mM KCl, 18 mM KH_2_PO_4_, and 100 mM Na_2_HPO_4_ (Sigma–Aldrich) at room temperature was injected to the left side of the heart for perfusion. Successful perfusion was confirmed by liver decoloration. Organs were excised and stored in ice-cold DMEM supplemented with 10% FCS, penicillin/streptomycin, 2 mM L-glutamine, and 1% MEM non-essential amino acids solution (Thermo Fisher Scientific).

### Tumor and thyroid dissociation

The fresh human ATC sample, subcutaneous tumors of NSG mice, and thyroids of Thyro-DT mice were washed with PBS, cut into small pieces, and enzymatically digested on a shaker for 60 minutes at 37°C in 5 mL RPMI-1640 supplemented with 1 mL 0.25% trypsin and 1 ml (0.1 g/mL) collagenase IV (Sigma–Aldrich). After digestion, supernatants were filtered through 70 μm cell strainers and washed with RPMI-1640, and cells were disaggregated by slowly pushing cell suspensions through 20G followed by 24G needles. Then, cells were washed, filtered through 40 μm cell strainers, and re-suspended in RPMI-1640 supplemented with 10% FCS.

### Flow cytometry (FACS)

ATC cell lines were cultured to 70% confluence and harvested using non-enzymatic cell dissociation buffer (Sigma–Aldrich). All stainings were performed in PBS for 20–30 minutes at 4°C. Details on antibodies, clones, manufacturers, and staining conditions for FACS are listed in Supplementary Table S3. Fixable Viability Dye-eFluor506 (dilution 1:4000) was from eBioscience. For mouse thyroid tumors, stained cells were washed and fixed using the BD Cytoperm/Cytofix kit (BD Biosciences) according to the manufacturer's instructions. Samples were acquired on a BD LSR II flow cytometer (Becton Dickinson) and were analysed using FlowJo software (TreeStar). Samples from NSG mice injected with GFP^hi^-expressing 8505C cells were also acquired on an ImageStreamX Mark II imaging flow cytometer and analyzed using IDEAS software (Amnis/EMD Millipore).

### Statistical analysis

Statistical analysis was performed using GraphPad Prism^®^ v5.0 (GraphPad Software). Data are represented as the mean ± standard error of the mean.

## Results

### Human ATCs express CD47 and are heavily infiltrated by macrophages

**FIG. 1.**
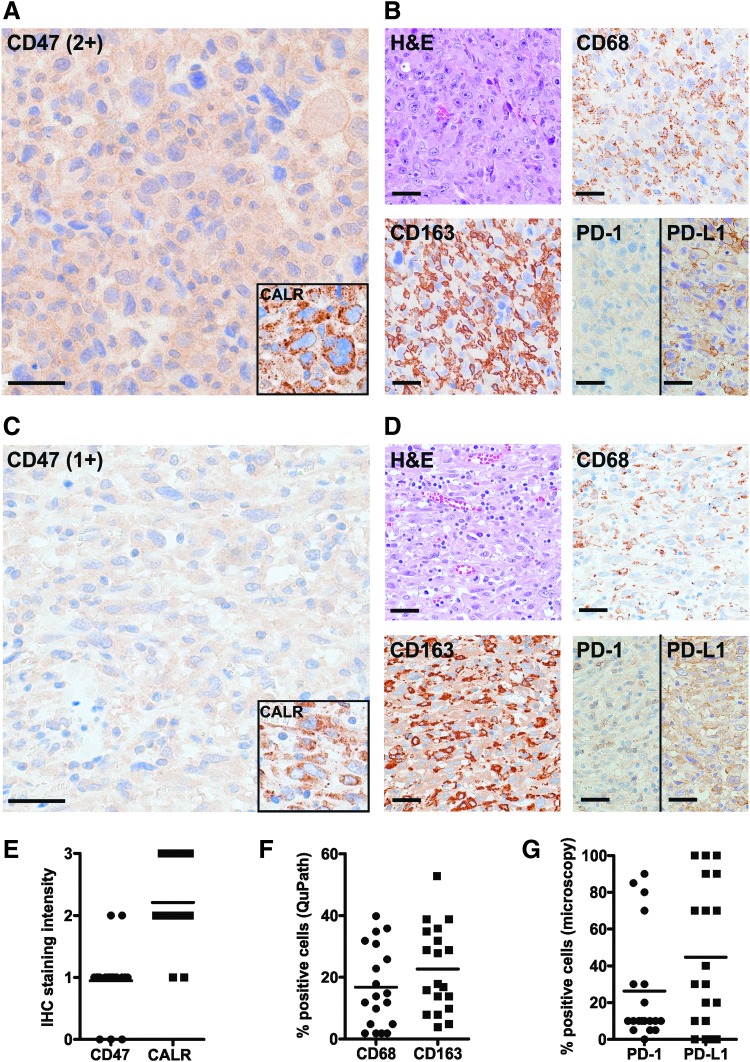
Human anaplastic thyroid carcinomas (ATCs) express phagocytosis signaling and immune checkpoint molecules and are heavily infiltrated by macrophages. Hematoxylin and eosin (H&E) staining and immunohistochemistry (IHC) for CD47, calreticulin (CALR), macrophage markers (CD68 and CD163), and checkpoint molecules (PD-1 and PD-L1) in ATCs. (**A** and **B**) Patient #8: epithelioid and sarcomatoid variant of ATC. CD47: 2+; CALR: 3+; CD68: 40% positive cells; CD163: 29% positive cells; PD-1: 5% positive cells; PD-L1: 70% positive cells. (**C** and **D**) Patient #14: epithelioid variant of ATC. CD47: 1+; CALR: 2+; CD68: 14% positive cells; CD163: 28% positive cells; PD-1: 10% positive cells; PD-L1: 70% positive cells. (**E**) IHC staining intensity of CD47 and CALR in ATCs, as analyzed by semi-quantitative microscopy. (**F**) Percentages of CD68^+^ cells and CD163^+^ cells in ATCs, as analyzed by automated quantification (QuPath). (**G**) Percentages of PD-1^+^ cells and PD-L1^+^ cells in all tumors, as analyzed by microscopy. Scale bars: 40 μm.

To study the role of CD47 and TAMs in ATC, FFPE tissues were used from a retrospective cohort of 19 patients who underwent surgery at the authors' hospital from 2005 to 2018. In this cohort, the median survival was 3.5 months (range 0.9–61 months). Patients were treated by either surgery alone or a combination of surgery, radiotherapy, and/or chemotherapy, as well as palliative supportive care (Table 1, Supplementary Table S1, and Supplementary Fig. S1). IHC revealed that most ATCs expressed low to moderate levels of CD47 in a cytoplasmic and/or membranous staining pattern (Fig. 1A, C, and E). Flow cytometry (FACS) analysis of a dissociated fresh ATC indicated strong surface CD47 expression in the CD45^−^ cell fraction containing the tumor cells (Supplementary Fig. S2). The pro-phagocytic molecule calreticulin was moderately to strongly expressed in a dot-like cytoplasmic pattern in most tumors analyzed by IHC (Fig. 1A, C, and E).

Previous studies have shown that ATCs are heavily infiltrated by TAMs (44–46). IHC was therefore performed for the macrophage markers CD68 and CD163 on the different ATC cases, which showed a mean macrophage infiltration rate of 17% (CD68) and 23% (CD163), as analyzed by automated digital quantification (Fig. 1B, D, and F and Supplementary Fig. S3). Similar results were obtained by FACS analysis of the dissociated fresh ATC tumor, with 13.5% of total cells being positive for CD68 (Supplementary Fig. S2). CD68 and CD163 percentages correlated moderately with each other; strong correlation was observed between automated quantification and semi-quantitative microscopic analysis for each macrophage marker (Supplementary Fig. S4).

Furthermore, PD-1 was expressed in ATCs on tumor cells, as well as on cells showing the histomorphological features of macrophages. Moreover, in line with previous studies (47–49), many tumors expressed high levels of the PD-1 ligand 1 (PD-L1; Fig. 1B, D, and G).

To address possible histological differences in patients with longer compared to shorter survival, the cohort was split into two groups: those surviving <6 vs. >12 months (*p* < 0.01). Tumor cell mitoses, CD68^+^ or CD163^+^ macrophages, CD47, calreticulin, or checkpoint molecules were not significantly different in these two groups (Supplementary Fig. S5). Furthermore, in-depth IHC analyses of the three most extreme outliers were performed (shortest- vs. longest-surviving patients with survival of <2 vs. >18 months, respectively). These results showed a trend toward higher percentages of T cells, lower tumor cell mitoses, and lower infiltration of CD15^+^ granulocytes and CD163^+^ macrophages in longer-surviving patients (Supplementary Fig. S6).

In summary, these data indicate that ATCs express the dominant pro-phagocytic molecule calreticulin, as well as the “don't eat me” signal CD47 and the immune checkpoint molecules PD-1 and PD-L1, all of which are currently targeted therapeutically in other types of cancer. In addition, they confirm that ATCs are infiltrated by innate and adaptive immune cells, pointing toward an important role of the immune microenvironment in these tumors.

### Blocking CD47 promotes the phagocytosis of human ATC cell lines by macrophages

**FIG. 2.**
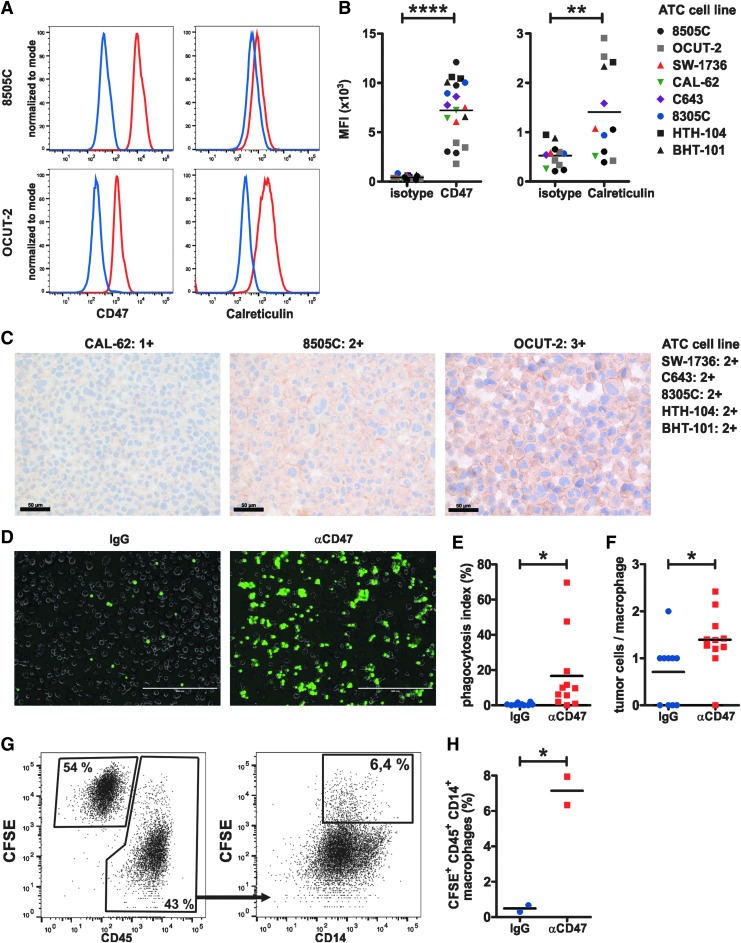
CD47 blockade promotes macrophage-mediated phagocytosis of human ATC cells. (**A**) Expression of CD47 and calreticulin on the ATC cell lines 8505C and OCUT-2, respectively, as analyzed by fluorescence-activated cell sorting (FACS). One representative histogram of three to four per staining is shown. Red lines, CD47 and calreticulin staining, respectively; blue lines, corresponding isotype controls. (**B**) Mean fluorescence intensity (MFI) of CD47 versus isotype (left panel) and calreticulin versus isotype (right panel) in eight different ATC cell lines. Pooled data from four independent experiments are shown. (**C**) IHC staining for CD47 on formalin-fixed, paraffin-embedded (FFPE) cell blocks from ATC cell lines. Scale bars: 50 μm. (**D–H**) *In vitro* phagocytosis assay. Peripheral blood mononuclear cell–derived macrophages were serum starved for two hours, followed by co-culture with 1 × 10^6^ 5(6)-carboxyfluorescein diacetate *N*-succinimidyl ester (CFSE)-labeled 8505C cells in the presence of 10 μg/mL IgG isotype control or anti-CD47 monoclonal antibody (mAb). (**D**) After two hours of co-culture, wells were thoroughly washed, and multiple fields of view (FOV) were imaged on an inverted fluorescence microscope. (**D**) Representative overlay images (one section of a FOV) for each condition are shown. Scale bars: 400 μm. (**E**) The percentage of macrophages with ingested CFSE^+^ tumor cells (phagocytosis index) and (**F**) the number of ingested tumor cells per macrophage were determined. Each dot represents a FOV (IgG: *n* = 3605 macrophages from 10 FOV; anti-CD47: *n* = 3371 macrophages from 11 FOV). One representative of two independent experiments is shown. (**G** and **H**) After microscopy, cells were dissociated, stained, and analyzed by FACS. (**G**) Gating strategy and (**H**) percentages of CFSE^+^ CD45^+^ CD14^+^ macrophages. Each dot represents one individual well of a six-well plate. One representative out of three independent experiments (each with two to three wells per condition) is shown. Statistics: (**B**) paired *t*-test, (**E**, **F**, and **H**) Student's *t*-test. *p < 0.05; **p < 0.01; ****p < 0.0001.

To investigate the effects of blocking anti-CD47 mAbs on phagocytosis of human ATC cell lines by macrophages *in vitro*, first the expression of CD47 and calreticulin was analyzed by FACS and IHC. Both CD47 and calreticulin were significantly expressed on the surface of all eight ATC cell lines analyzed (Fig. 2A–C). In addition to membranous staining, IHC revealed weak cytoplasmic CD47 staining in most cell lines (Fig. 2C). Next, human macrophages were co-cultured with CFSE-labeled 8505C ATC cells in the presence of anti-CD47 or isotype control mAb. Inverted fluorescence microscopy of co-cultures (Fig. 2D) revealed that blocking CD47 significantly increased the numbers of macrophages with phagocytosed ATC cells (phagocytosis index, Fig. 2E), as well as the number of phagocytosed ATC cells per macrophage (Fig. 2F). These results were further validated by FACS analysis of the co-cultures (Fig. 2G and H). Thus, blocking CD47 promotes the phagocytosis of human ATC cells by macrophages.

### Blocking CD47 inhibits ATC growth and increases TAMs and tumor cell phagocytosis *in vivo*

**FIG. 3.**
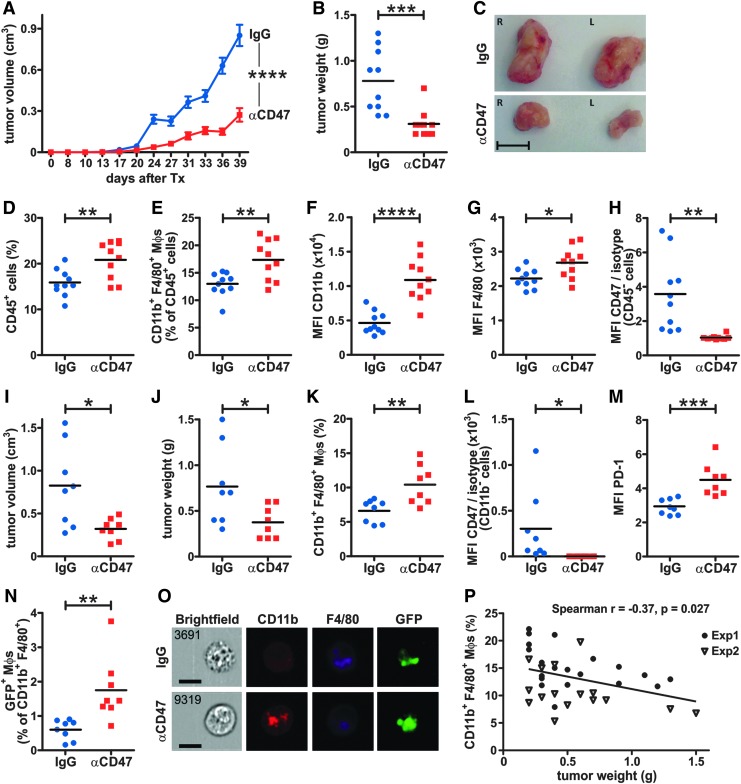
CD47 blockade increases tumor-associated macrophage (TAM) frequency, promotes phagocytosis, and inhibits ATC growth *in vivo*. (**A–H**) 8505C cells (5 × 10^6^) were injected subcutaneously (s.c.) into each flank of NSG mice. After three days, mice were treated with 500 μg anti-CD47 mAb (*n* = 5) or IgG isotype control mAb (*n* = 5) intraperitoneally (i.p.) twice a week for six weeks and (**A**) tumor volumes were monitored using a caliper at the indicated time points after injection. (**B**) Tumor weights and (**C**) representative images of tumors at the endpoint. Scale bar: 1 cm. (**D–H**) Tumors were dissociated and analyzed by FACS. (**D** and **E**) The frequencies of tumor-infiltrating (**D**) total CD45^+^ cells and (**E**) CD11b^+^ F4/80^+^ macrophages as well as the MFIs of (**F**) CD11b and (**G**) F4/80 expression on CD11b^+^ F4/80^+^ macrophages were determined. (**H**) MFI of CD47 staining versus isotype control on CD45^−^ tumor cells of IgG- and anti-CD47-treated mice. CD47 staining was performed using the same mAb clone as for treatment. (**I–O**) Stably GFP-expressing 8505C cells (4 × 10^6^) were injected s.c. into each flank of NSG mice. After three days, mice were treated with 500 μg anti-CD47 mAb (*n* = 4) or IgG isotype control mAb (*n* = 4) i.p. twice a week. Five weeks later, tumors were excised, and (**I**) tumor volumes and (**J**) tumor weights were measured. (**K**) The frequency of CD11b^+^ F4/80^+^ macrophages and (**L**) MFI of CD47 staining versus isotype control on CD11b^−^ tumor cells. (**M**) The MFI of PD-1 expression on CD11b^+^ F4/80^+^ macrophages as well as (**N**) the frequency of GFP^+^ CD11b^+^ F4/80^+^ macrophages were determined. (**O**) ImageStream^®^ analysis of GFP^+^ CD11b^+^ F4/80^+^ macrophages from IgG- and anti-CD47-treated mice. One representative image of *n* = 25 (anti-CD47) and *n* = 10 (IgG) cells from two tumors per treatment group is shown. Scale bars: 10 μm. (**P**) Correlation of tumor weights and the frequencies of tumor-infiltrating CD11b^+^ F4/80^+^ macrophages in both experiments. Exp1, data from (**A**–**H**); exp2, data from (**I**–**O**). Statistics: (**A**) two-way analysis of variance, (**B** and **D**–**N**) Student's *t*-test, (**P**) two-tailed *t*-test. *p < 0.05; **p < 0.01; ***p < 0.001; ****p < 0.0001.

To validate the *in vitro* phagocytosis assays *in vivo*, xenotransplantation studies were performed by injecting 8505C cells subcutaneously into the flanks of NOD.Cg-*Prkdc^scid^ Il2rg^tm1WjI^*/SzJ (NSG) mice. After three days of engraftment, mice were treated with blocking anti-human CD47 mAb or IgG isotype control twice a week. Blocking CD47 resulted in significantly reduced tumor volumes and weights after six weeks of treatment (Fig. 3A–C). Frequencies of CD45^+^ tumor-infiltrating leukocytes and CD11b^+^ F4/80^+^ double-positive (DP) macrophages were significantly higher in anti-CD47-treated mice (Fig. 3D and E). Interestingly, the expression of CD11b integrin and F4/80 was also significantly increased in TAMs from anti-CD47-treated mice compared to controls (Fig. 3F and G), suggesting that blocking CD47 activates macrophage function. Importantly, the lack of signal on CD45^−^ tumor cells upon CD47 staining using the same mAb clone as for treatment indicated that the CD47 blockade was effective *in vivo* (Fig. 3H).

To investigate in more detail whether anti-CD47 treatment indeed improves phagocytosis by the accumulated TAMs, stably GFP-expressing 8505C cells were xenotransplanted into NSG mice. Tumor volumes and weights (Fig. 3I and J), the frequency of DP macrophages in tumors (Fig. 3K), and the blocking of CD47 on GFP-expressing 8505C cells (Fig. 3L) were comparable to conditions using parental 8505C cells. Interestingly, a higher PD-1 expression was observed on DP macrophages from anti-CD47-treated mice (Fig. 3M). More importantly, the frequency of GFP-expressing DP macrophages was significantly higher in anti-CD47-treated mice compared to controls, as analyzed by FACS (Fig. 3N). This was confirmed by ImageStream^®^ analysis showing GFP^+^ cellular debris inside DP macrophages (Fig. 3O). In addition, tumor weights in both experiments inversely correlated with the frequency of DP macrophages in tumors (Fig. 3P), indicating that tumor weight and volume in xenotransplanted mice were substantially affected by the presence of infiltrating macrophages.

In summary, these results indicate that anti-CD47 treatment promotes the accumulation of activated TAMs and improves their phagocytic function in ATC tumors *in vivo*.

### Blocking CD47 increases TAMs in a transgenic ATC mouse model

**FIG. 4.**
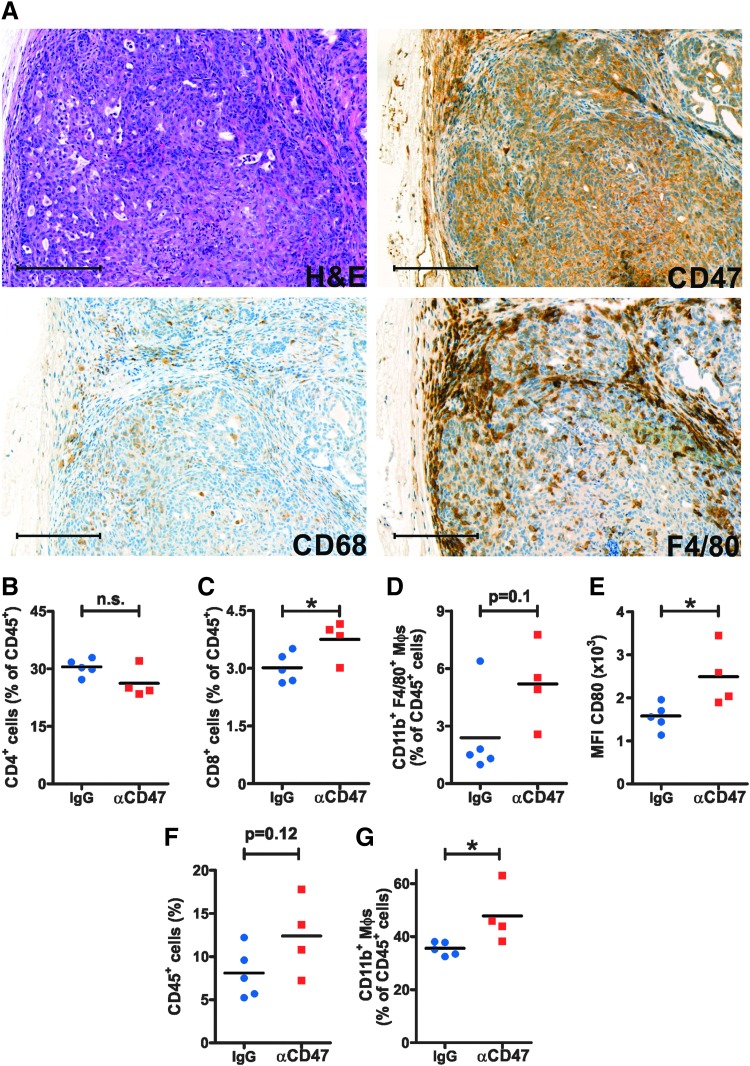
CD47 blockade promotes TAM accumulation in a transgenic mouse model of ATC. (**A**) H&E staining and IHC for CD47, CD68, and F4/80 in FFPE tumor samples from Thyro-DT mice. One representative image of tumors from eight mice is shown for each staining. Scale bars: 200 μm. (**B–G**) Three months after transgene induction, tumor-bearing Thyro-DT mice were divided in two groups with similar tumor size and treated with either 500 μg anti-CD47 mAb (*n* = 4) or antibody dilution buffer (*n* = 5) by i.p. injection twice a week. Tumor growth was measured by ultrasound every two weeks and normalized to baseline before start of treatment. After 10 weeks of treatment, mice were sacrificed, and spleens and thyroids were analyzed by FACS. (**B–D**) Frequencies of (**B**) CD4^+^ T cells, (**C**) CD8^+^ T cells, and (**D**) CD11b^+^ F4/80^+^ macrophages were determined in the spleen. (**E**) MFI of CD80 expression on splenic CD11b^+^ F4/80^+^ macrophages. (**F** and **G**) The frequencies of tumor-infiltrating (**F**) total CD45^+^ cells and (**G**) CD11b^+^ macrophages were determined in the thyroids of Thyro-DT mice. Data from one thyroid lobe per mouse are shown. Statistics: Student's *t*-test. *p < 0.05. n.s., not significant.

To study the effects of anti-CD47 treatment in a more clinically relevant setting, we made use of the *Braf^CA/+^; Pik3ca^Lat/+^; ThyroglobulinCre^ERT2^* (Thyro-DT) mouse model (43). Thyro-DT mice start developing fatal high-grade thyroid carcinomas between three and six months after transgene induction with tamoxifen. First, the expression of CD47 and macrophage markers was analyzed in FFPE thyroid tissues from tumor-bearing Thyro-DT mice. Neoplastic epithelial cells showed intermediate to strong expression of CD47 (Fig. 4A). Staining for CD68 and F4/80 revealed numerous TAMs in the neoplastic epithelium and tumor stroma (Fig. 4A). Three months after tamoxifen induction, tumor-bearing mice were divided into two groups with similar tumor sizes, as measured by ultrasound. Mice were treated with either anti-CD47 mAb or antibody dilution buffer, respectively, and subsequent tumor growth was repeatedly measured by ultrasound. During the 10-week treatment period, mouse tumors nearly doubled in size. Yet, no significant difference in tumor size was observed between the two groups (data not shown). However, FACS analysis of spleens revealed that anti-CD47 treatment had induced significantly increased frequencies of CD8^+^ T cells and a trend toward higher frequencies of DP macrophages. Proportions of splenic CD4^+^ T cells remained comparable between the two experimental groups (Fig. 4B–D). Interestingly, the expression of CD80 on DP macrophages in the spleen was significantly increased after CD47 blockade (Fig. 4E). In thyroids, there was a trend toward higher frequencies of total tumor-infiltrating CD45^+^ cells, and the frequency of CD11b^+^ macrophages was significantly increased (Fig. 4F and G). FACS analysis using anti-mouse immunoglobulin revealed that the therapeutic CD47 mAb had effectively bound on the surface of CD45^−^ EpCAM^+^ tumor cells (data not shown).

These data indicate that murine ATCs are infiltrated by macrophages *in vivo* and suggest that CD47 blockade modulates the composition of the tumor immune infiltrate as well as systemic immunity in transgenic ATC-bearing mice.

## Discussion

ATC is a rare, aggressive tumor for which effective therapeutic options are limited. Many ATCs harbor one or multiple recurrent genetic mutations that are potentially druggable (50). Targeted therapy approaches that have been reported or are currently being tested clinically, alone or in combination with chemotherapy, include *BRAF* inhibitors (51), *BRAF*/*MEK* inhibitors (Clinicaltrials.gov identifier NCT02034110) (52), mechanistic target of rapamycin (mTOR) inhibitors (NCT02244463, NCT00936858) (53), improved multi-kinase inhibitors (54), novel anti-microtubule agents (55), peroxisome proliferator-activated receptor-γ (PPAR-γ) agonists (56), combined histone deacetylase (HDAC) and phosphoinositide 3-kinase (PI3K) inhibitors (NCT03002623), and anaplastic lymphoma kinase (ALK) inhibitors (57). However, most of these small-molecule therapies only work in subgroups of ATCs with the respective driver mutations or pathway alterations. In addition, the plasticity of cancer cells in general often rapidly leads to therapy-induced drug resistance under single-agent small molecule treatments by mechanisms such as drug target mutation or alternative activation of the targeted pathways (58). Therefore, such treatments usually fail to induce long-lasting remissions.

In contrast to the above-mentioned mutations, most of which are only present in subgroups of ATC (50), most ATC tumors are infiltrated by TAMs and T cells and express CD47 and PD-L1 (44–47,49). Moreover, compared to small molecule inhibitors, immunotherapy—particularly the blockade of PD-1, PD-L1, or CTLA-4—is inherently multivalent because a single drug unleashes multi-specific antitumoral T-cell responses (59). Resistance mechanisms to immunotherapy have also been observed and are related to diverse factors, including the tumor microenvironment, patient age, hormonal levels, and the microbiome (60). Nevertheless, immune checkpoint inhibitors have shown exceptional efficacy and have led to long-lasting remissions in subgroups of patients harboring a broad array of advanced and even metastatic solid tumors, as well as hematological cancers (61,62).

For ATC, clinical immunotherapy trials that investigate immune checkpoint inhibitory mAbs against PD-1 (NCT02721732, NCT02404441) or a combination of anti-CTLA-4 + anti-PD-1 mAbs (NCT03246958) are currently under way.

In addition to checkpoint inhibitors that mainly act by increasing the function of effector T cells (59), recent research activities have focused on antitumoral mediators of the innate immune system, mainly NK cells and macrophages (15,63,64). Hitherto, TAMs have generally been labeled as a pro-tumorigenic cell population. M2-polarized TAMs produce cytokines and chemokines that directly promote tumor growth, induce angiogenesis and tumor cell invasion, and suppress effective adaptive immune responses against tumors (65,66). Concordantly, in the present cohort, patients with long survival (i.e., >12 months, including a patient who is still alive 61 months after diagnosis) generally showed lower numbers of TAMs that express the scavenger receptor CD163, a marker for M2 macrophages (Supplementary Fig. S5) (67). Attempts to eliminate TAMs using mAbs against colony stimulating factor 1 (CSF-1) or small molecule inhibitors targeting the c-fms tyrosine kinase of its receptor (CSF-1R/CD115) have proven effective in various murine cancer models and are investigated in numerous clinical trials (68,69).

On the other hand, instead of removing M2-polarized TAMs from tumors, reprogramming them toward an antitumoral, inflammatory M1 (classical) phenotype is an attractive therapeutic strategy (66). Classically activated TAMs stimulated by bacterial products (70) or anti-CD40 mAbs (71) effectively inhibit tumor growth. Because TAMs form a substantial fraction of total cells in most ATCs, we hypothesize that their activation against tumor cells represents a powerful strategy. We found that CD47 blockade *in vivo* induced an upregulation of CD11b, F4/80, and CD80 on TAMs and splenic macrophages, respectively, which is consistent with macrophage activation. Moreover, in ATC xenotransplanted NSG mice, anti-CD47 mAb treatment increased TAM frequencies and promoted phagocytosis of tumor cells, resulting in significantly delayed tumor growth. Correspondingly, blocking CD47 increased TAM frequencies in transgenic Thyro-DT mice compared to controls. However, in this model, differences in tumor growth were not observed. Furthermore, in ATC xenotransplanted NSG mice, CD47 blockade increased PD-1 expression in TAMs. Besides the well-established role for PD-1 in the suppression of adaptive antitumoral T-cell responses (9), it was shown that TAMs express PD-1, which inhibits their phagocytosis and thereby also prevents innate antitumoral immunity (72). Therefore, future studies should address co-inhibition of CD47/SIRPα and PD-1/PD-L1 in ATC. This combination will likely further enhance TAM activity and tumor cell phagocytosis and induce synergistic antitumoral CD8^+^ T-cell responses (47,49). Concordantly, Liu *et al*. recently demonstrated that treatment with a bispecific anti-PD-L1/SIRPα mAb induced effective, combined adaptive and innate antitumoral immune responses against PD-L1- and CD47-expressing solid tumors, including colorectal cancer and melanoma (73).

In summary, our work provides evidence that the “don't eat me” signal CD47 is expressed on human ATC to prevent TAM-mediated tumor phagocytosis. Our findings also suggest that anti-CD47 alone or combined with anti-PD-1 mAb immunotherapies may represent a valuable addition to the current standard of care for ATC patients.

## Supplementary Material

Supplemental data

Supplemental data

Supplemental data

Supplemental data

Supplemental data

Supplemental data

Supplemental data

Supplemental data

Supplemental data
