# Supplementary material for: Targeting CD47 in Anaplastic Thyroid Carcinoma Enhances Tumor Phagocytosis by Macrophages and Is a Promising Therapeutic Strategy
Source: Thyroid. 2019 Jul 17;29(7):979–92. doi: 10.1089/thy.2018.0555 (PMC6648226; doi:10.1089/thy.2018.0555)
Supplement: Supplemental data [file Supp_Table2.pdf]

Supplementary Table S2. Antibodies, Clones, Manufacturers, and Staining Conditions for IHC

Antigen	*Reactivity*	*Host*	*Clone*	*Manufacturer*	*Dilution*	*Pretreatment*
Calponin	Human	Mouse	CALP1	Dako	1:800	Protease 37°C, 5 min
Calreticulin	Human	Mouse	FMC75	Abcam	1:6000	Citrate buffer 100°C, 30 min
CD3	Human	Mouse	LN10	Novocastra	1:200	EDTA buffer 100°C, 20 min
CD4	Human	Mouse	4B12	Novocastra	1:100	EDTA buffer 100°C, 30 min
CD8	Human	Mouse	C8/144B	Cell Marque	1:200	EDTA buffer 100°C, 20 min
CD15	Human	Mouse	MMA	BD Biosciences	1:25	EDTA buffer 95°C, 40 min
CD31	Human	Mouse	JC/70A	Dako	1:800	EDTA buffer 95°C, 30 min
CD47	Human	Mouse	B6H12	Santa Cruz	1:20	Tris buffer 95°C, 30 min
CD47	Mouse	Goat	poly	R&D Systems	1:750	Tris buffer 95°C, 30 min
CD56	Human	Mouse	CD564	Novocastra	1:400	EDTA buffer 100°C, 20 min
CD68	Human	Mouse	PG-M1	Dako	1:100	EDTA buffer 100°C, 30 min
CD68	Mouse	Rat	FA11	ABD Serotec	1:100	Citrate buffer 100°C, 30 min
CD123	Human	Mouse	BR4MS	Novocastra	1:100	EDTA buffer 95°C, 40 min
CD163	Human	Mouse	10D6	Novocastra	1:400	EDTA buffer 95°C, 20 min
F4/80	Mouse	Rat	A3-1	ABD Serotec	1:50	Protease 37°C, 5 min
Fibroblast activation protein	Human	Mouse	1E5	Abcam	1:1500	Tris buffer 95°C, 30 min
Ki-67	Human	Rabbit	SP6	Cell Marque	1:100	EDTA buffer 95°C, 40 min
PD-1	Human	Mouse	NAT-105	Cell Marque	1:150	Tris buffer 95°C, 30 min
PD-L1	Human	Rabbit	E1L3N	Cell Signaling Technology	1:400	Tris buffer 95°C, 40 min
Smooth muscle actin	Human	Mouse	1A4	Sigma	1:8000	—
Vimentin	Human	Mouse	3B4	Dako	1:1600	EDTA buffer 95°C, 20 min
