# Supplementary material for: Targeting CD47 in Anaplastic Thyroid Carcinoma Enhances Tumor Phagocytosis by Macrophages and Is a Promising Therapeutic Strategy
Source: Thyroid. 2019 Jul 17;29(7):979–92. doi: 10.1089/thy.2018.0555 (PMC6648226; doi:10.1089/thy.2018.0555)
Supplement: Supplemental data [file Supp_Table3.pdf]

Supplementary Table S3. Antibodies, Clones, Manufacturers, and Staining Conditions for FACS

Antigen	*Reactivity*	*Host*	*Clone*	*Manufacturer*	*Dilution*	*Conjugate*
CD4	Mouse	Rat	RM4-5	BioLegend	1:1000	APC-Cy7
CD8a	Mouse	Rat	53-6.7	BioLegend	1:200	FITC
CD11b	Mouse/human	Rat	M1/70	BioLegend	1:400	Pacific Blue
CD14	Human	Mouse	M5E2	BioLegend	1:20	APC
CD45	Human	Mouse	HI30	BioLegend	1:50	Pacific Blue
CD45	Mouse	Rat	30-F11	BioLegend	1:200	Alexa Fluor 700
CD47	Human	Mouse	CC2C6	BioLegend	1:10	FITC
isotype control	Unknown	Mouse	P3.6.2.8.1	eBioscience	1:10	FITC
CD47	Human	Mouse	B6H12	eBioscience	1:20	APC
isotype control	Unknown	Mouse	P3.6.2.8.1	eBioscience	1:20	APC
Calreticulin	Human	Mouse	FMC75	Abcam	1:50	PE
isotype control	Unknown	Mouse	B11/6	Abcam	1:1.25	PE
CD68	Human	Mouse	Y1/82A	BD Biosciences	1:20	PE
Mouse Ig	Mouse	Goat	polyclonal	BD Biosciences	1:200	PE
CD80	Mouse	Rat	16-10A1	BioLegend	1:100	PerCP-Cy5.5
CD326 (EpCAM)	Mouse	Rat	G8.8	BioLegend	1:1000	PE-Cy7
F4/80	Mouse	Rat	BM8	eBioscience	1:400	APC
