# Supplementary material for: Targeting CD47 in Anaplastic Thyroid Carcinoma Enhances Tumor Phagocytosis by Macrophages and Is a Promising Therapeutic Strategy
Source: Thyroid. 2019 Jul 17;29(7):979–92. doi: 10.1089/thy.2018.0555 (PMC6648226; doi:10.1089/thy.2018.0555)
Supplement: Supplemental data [file Supp_Fig2.pdf]

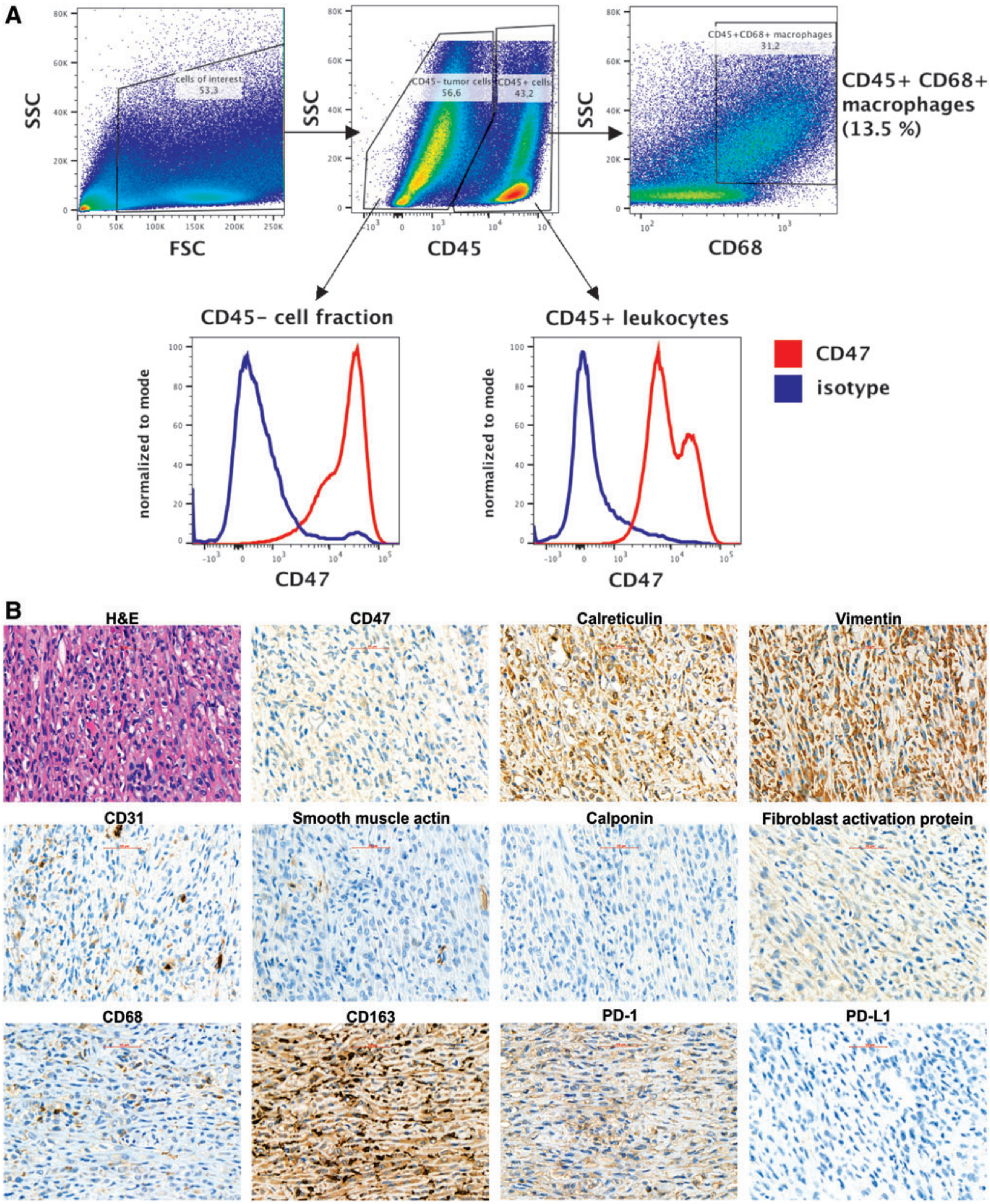

SUPPLEMENTARY FIG. S2. FACS analysis of CD47 and CD68 expression in human ATC tissue. (**A**) After frozen section diagnosis, four small pieces of fresh tumor from different regions were dissociated and stained with CD45, CD68, CD47, and isotype control antibodies. One representative sample of four different tumor regions is shown. Red lines, CD47 staining; blue lines, isotype control. (**B**) A representative FFPE block from the same tumor was stained by H&E and IHC for CD47 and calreticulin, cytoplasmic intermediate filaments (vimentin), endothelial cells (CD31), smooth-muscle cells (smooth-muscle actin, calponin), fibroblasts (fibroblast activation protein), macrophages (CD68, CD163), and checkpoint markers (PD-1, PD-L1). Scale bars: 50 μm.
