# Supplementary material for: Targeting CD47 in Anaplastic Thyroid Carcinoma Enhances Tumor Phagocytosis by Macrophages and Is a Promising Therapeutic Strategy
Source: Thyroid. 2019 Jul 17;29(7):979–92. doi: 10.1089/thy.2018.0555 (PMC6648226; doi:10.1089/thy.2018.0555)
Supplement: Supplemental data [file Supp_Fig3.pdf]

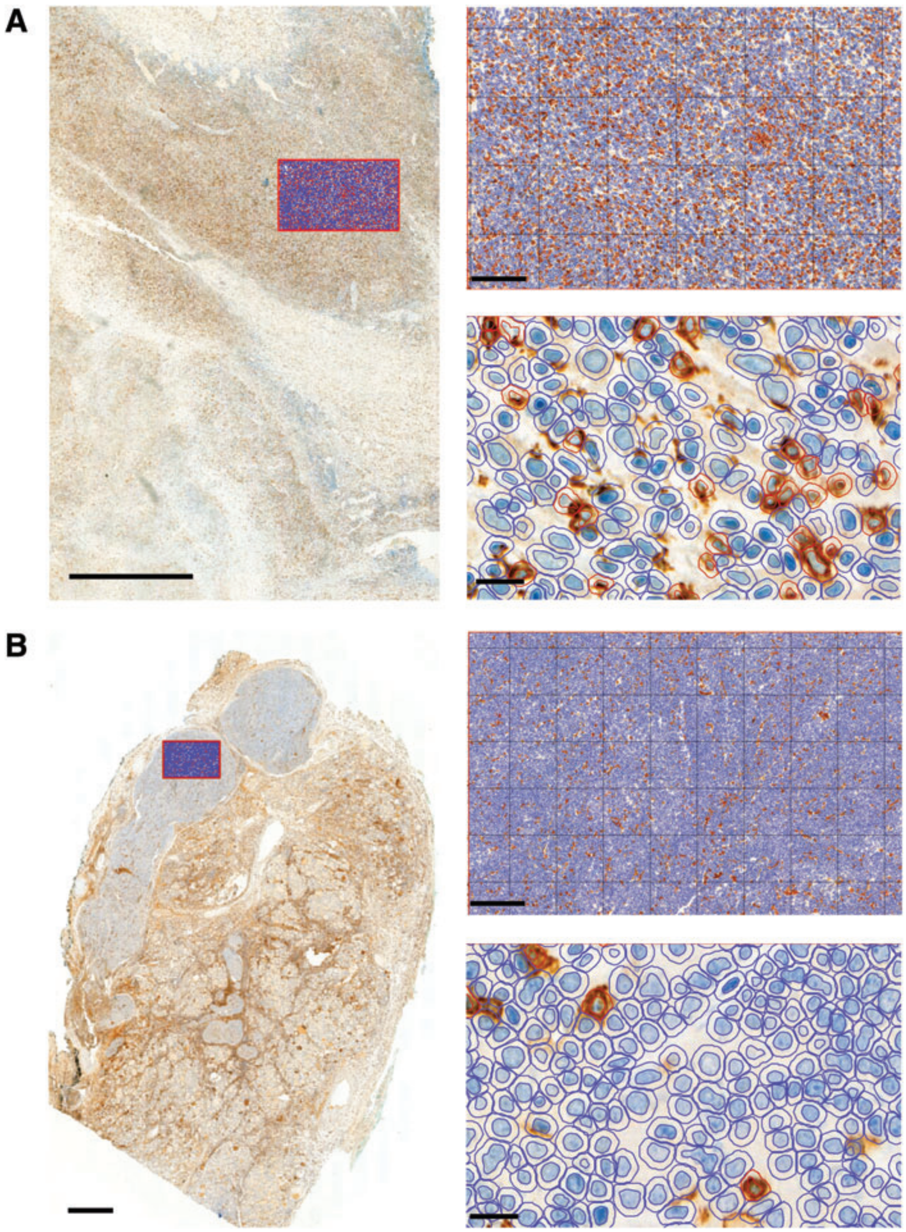


**SUPPLEMENTARY FIG. S3.** Example images of QuPath automated segmentation results. (**A**) CD68 IHC staining (patient 16) and (**B**) CD163 IHC staining (patient 3). Scale bars: 2 mm, 200 $\mu$m, and 20 $\mu$m for the different magnifications, respectively.
