# Supplementary material for: Targeting CD47 in Anaplastic Thyroid Carcinoma Enhances Tumor Phagocytosis by Macrophages and Is a Promising Therapeutic Strategy
Source: Thyroid. 2019 Jul 17;29(7):979–92. doi: 10.1089/thy.2018.0555 (PMC6648226; doi:10.1089/thy.2018.0555)
Supplement: Supplemental data [file Supp_Fig4.pdf]

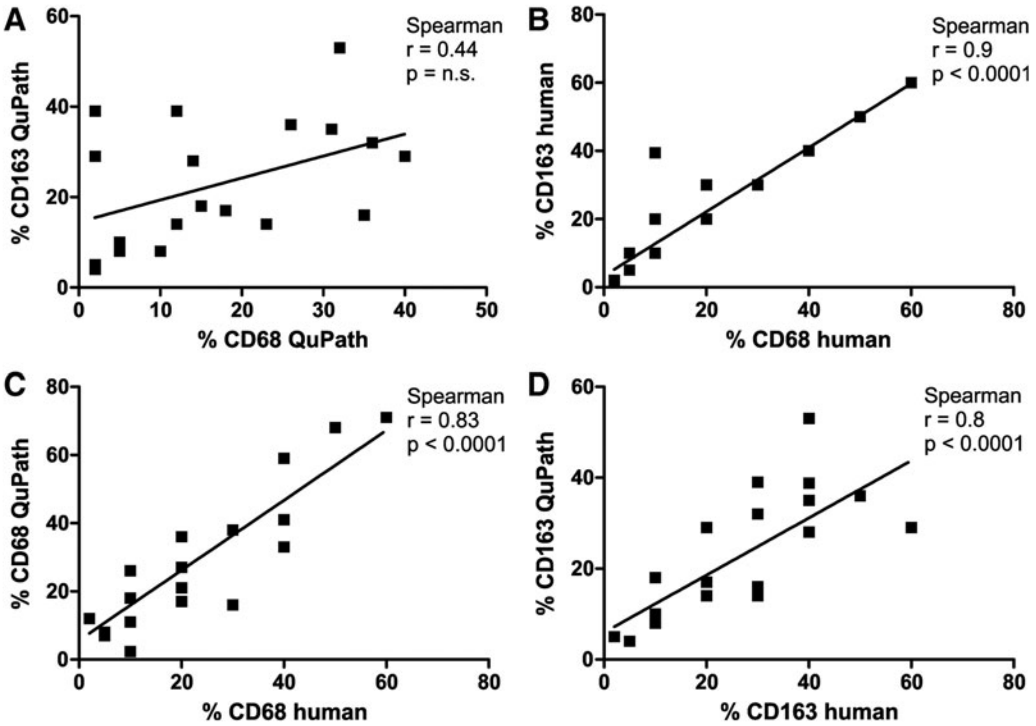

SUPPLEMENTARY FIG. S4. CD68 and CD163 expression correlate in ATC tumors. (**A** and **B**) Correlation of percentages of $CD68^+$ and $CD163^+$ cells as analyzed by (**A**) QuPath automated segmentation or (**B**) semi-quantitative analysis by microscopy. (**C** and **D**) Correlation of QuPath automated segmentation versus semi-quantitative microscopic analysis for the percentages of (**C**) $CD68^+$ cells and (**D**) $CD163^+$ cells.
