# Supplementary material for: Targeting CD47 in Anaplastic Thyroid Carcinoma Enhances Tumor Phagocytosis by Macrophages and Is a Promising Therapeutic Strategy
Source: Thyroid. 2019 Jul 17;29(7):979–92. doi: 10.1089/thy.2018.0555 (PMC6648226; doi:10.1089/thy.2018.0555)
Supplement: Supplemental data [file Supp_Fig5.pdf]

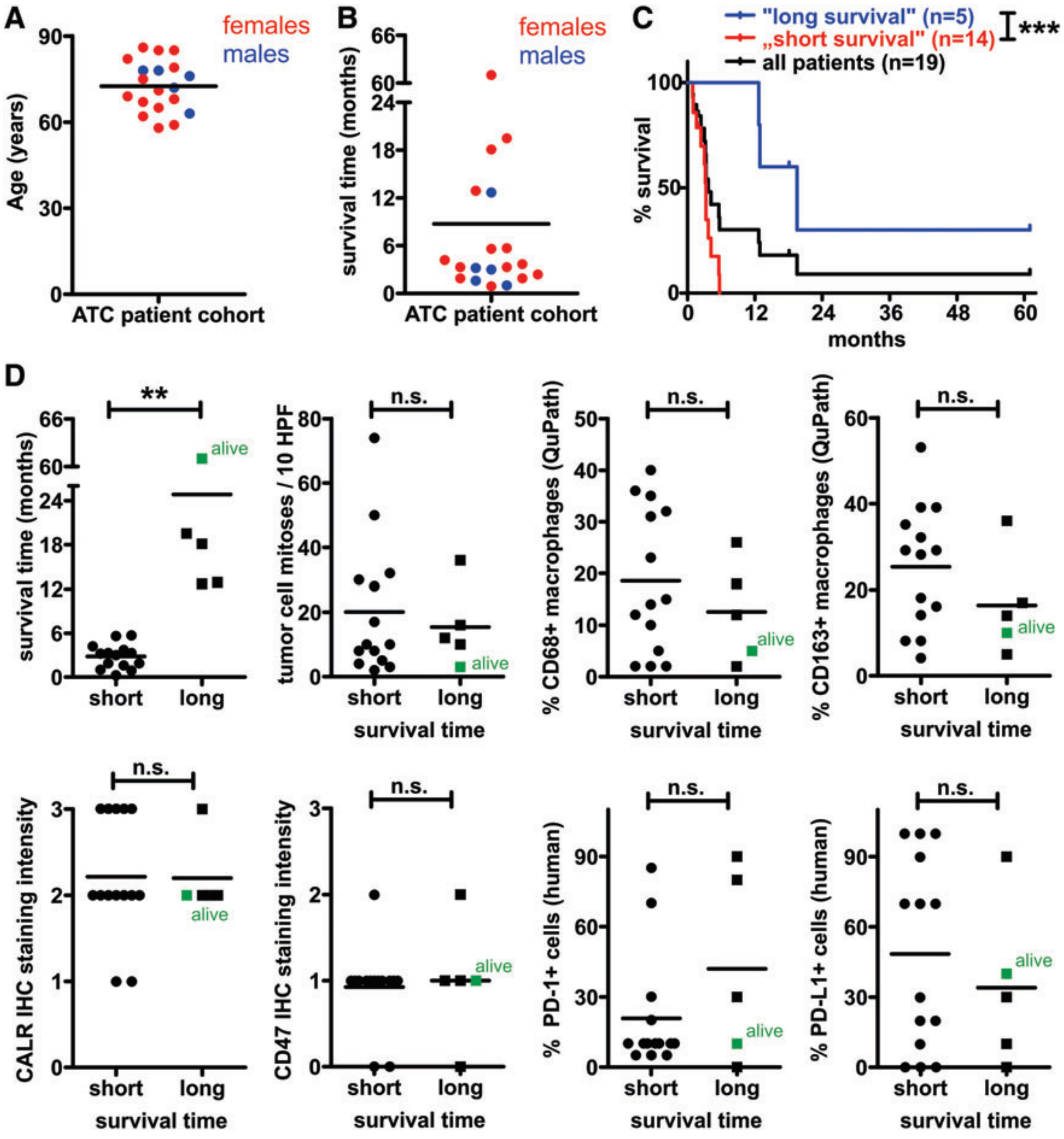

SUPPLEMENTARY FIG. S5. Survival subgroups analysis. (**A**) Age and sex and (**B**) survival time distribution in the entire ATC cohort. (**C**) Kaplan–Meier survival curves for all patients and the ''short''- versus ''long''-surviving patients. (**D**) Survival time, tumor cell mitoses, $CD68^+$ macrophages, $CD163^+$ macrophages, calreticulin staining, CD47 staining, $PD\text{-}1^+$ cells, and $PD\text{-}L1^+$ cells in the two different survival subgroups. Statistics: (**C**) log-rank test; (**D**) Mann–Whitney *U*-test. $^{**}p<0.01$; $^{***}p<0.001$. n.s., not significant.
