# Supplementary material for: Targeting CD47 in Anaplastic Thyroid Carcinoma Enhances Tumor Phagocytosis by Macrophages and Is a Promising Therapeutic Strategy
Source: Thyroid. 2019 Jul 17;29(7):979–92. doi: 10.1089/thy.2018.0555 (PMC6648226; doi:10.1089/thy.2018.0555)
Supplement: Supplemental data [file Supp_Fig6.pdf]

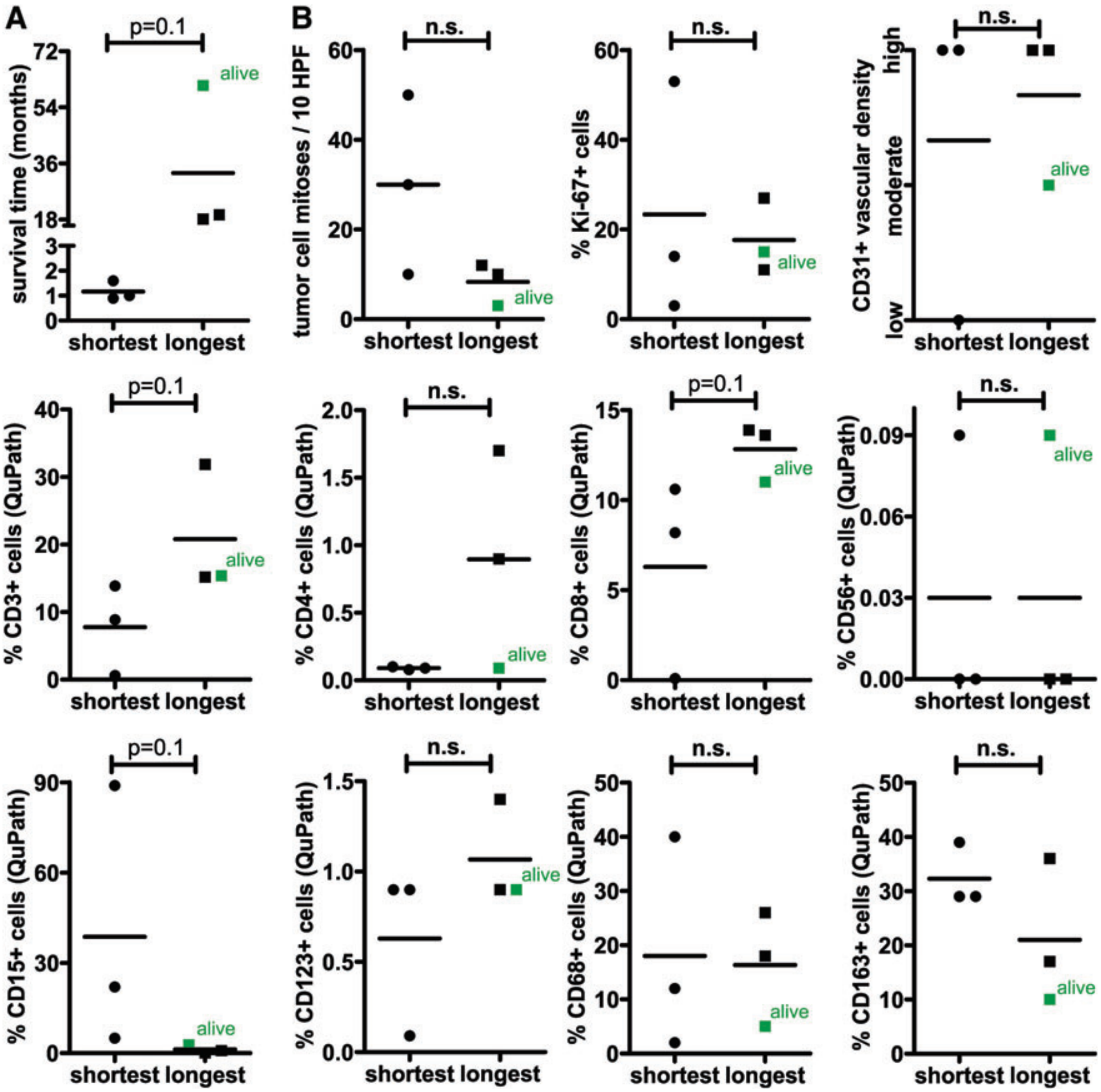

SUPPLEMENTARY FIG. S6. In-depth IHC analysis. The immune infiltrate in three most extreme survival outliers of each subgroup was further analyzed. (**A**) Survival time. (**B**) Tumor cell mitoses, Ki-67$^+$ cells, CD31$^+$ vascular density, CD3$^+$, CD4$^+$, and CD8$^+$ T cells, CD56$^+$ NK cells, CD15$^+$ granulocytes, CD123$^+$ plasmacytoid dendritic cells, CD68$^+$ macrophages, and CD163$^+$ macrophages in the outliers. Statistics: Mann–Whitney *U*-test. n.s., not significant.
